# Analysis of a *C. elegans* lifespan prediction method based on a bimodal neural network and uncertainty estimation^☆^

**DOI:** 10.1016/j.csbj.2022.12.033

**Published:** 2022-12-29

**Authors:** Antonio García-Garví, Pablo E. Layana-Castro, Antonio-José Sánchez-Salmerón

**Affiliations:** Instituto de Automática e Informática Industrial, Universitat Politècnica de València, Camino de Vera S/N, Valencia 46022, Spain

**Keywords:** *C. elegans*, Lifespan prediction, Deep learning, Synthetic data, Multimodal neural network

## Abstract

In recent decades, assays with the nematode *Caenorhabditis elegans* (*C. elegans*) have enabled great advances to be made in research on aging. However, performing these assays manually is a laborious task. To solve this problem, numerous *C. elegans* assay automation techniques are being developed to increase throughput and accuracy. In this paper, a method for predicting the lifespan of *C. elegans* nematodes using a bimodal neural network is proposed and analyzed. Specifically, the model uses the sequence of images and the count of live *C. elegans* up to the current day to predict the lifespan curve termination. This network has been trained using a simulator to avoid the labeling costs of training such a model. In addition, a method for estimating the uncertainty of the model predictions has been proposed. Using this uncertainty, a criterion has been analyzed to decide at what point the assay could be halted and the user could rely on the model’s predictions. The method has been analyzed and validated using real experiments. The results show that uncertainty is reduced from the mean lifespan and that most of the predictions obtained do not present statistically significant differences with respect to the curves obtained manually.

## Highlights

• A new method is proposed for predicting lifespan curves using a bimodal neural network trained with synthetic data.
• A method is put forward for estimating uncertainty in model predictions.
• A criterion was defined and analyzed in order to halt the assay.

## Graphical Abstract

**Figure:**
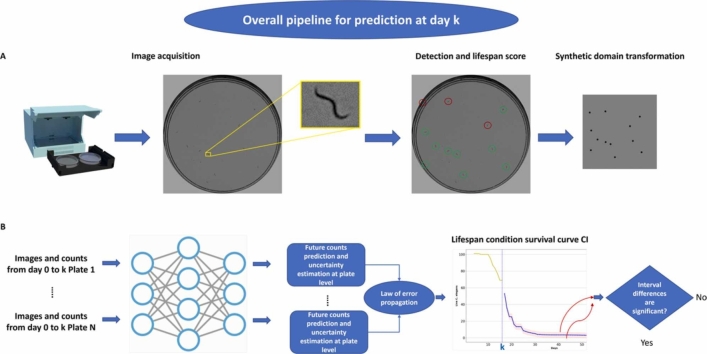

## 1 Introduction

In recent decades the life expectancy of the human population has increased. However, this increase in life expectancy does not translate into a better quality of life. With aging, the probability of suffering from neurodegenerative diseases such as Alzheimer’s or Parkinson’s increases. This has led the scientific community to take a great interest in understanding the mechanisms that regulate aging, as well as in the search for new drugs to help alleviate these diseases. The nematode *Caenorhabditis elegans* (*C. elegans*) is one of the most widely used model animals in aging studies due to its characteristics [1]: namely, a short life span (approximately 3 weeks); a small size, facilitating cultivation of large populations; and a transparent body, making organs and tissues visible under the microscope. Furthermore, its genomic sequence is known [2] and between 60 % and 80 % are homologues with humans. Research using this nematode model has led to major advances in the understanding of the mechanisms that regulate aging [3], [4], [5].

The lifespan and healthspan assays are the gold standard in *C. elegans* aging research. On the one hand, lifespan assays evaluate the survival curve of a population subjected to certain conditions and compare it with a control group to determine the influence of the conditions on population survival rates [6]. On the other hand, healthspan [7] assays seek to measure the age to which the population remains healthy. These laborious and time-consuming experiments are performed manually in most laboratories. This prevents the full potential of the assays from being realized, as the number of conditions and worms that can be tested simultaneously is limited. For this reason, automation of these assays is essential to increase productivity, as well as to obtain new and more accurate measurements. Such measurements allow the estimation of lifespan and healthspan, and previous studies have also claimed that mid-phase activity is a good estimator of lifespan. With respect to previous publications: In Ref. [8] identified the rate of deterioration of motor activity in the early and middle phase of aging as an endogenous physiological parameter that can predict lifespan; In Ref. [9] showed that maximum velocity, if measured in mid-adulthood, predicts maximum lifespan; Martineau et al. [10] predicted the age, remaining life and lifespan of each *C. elegans* using support vector machine (SVM) from hundreds of morphological, postural and behavioral features extracted from high-resolution videos. More recently, metrics based on the rate of decline in collective activity have been used to estimate the average lifespan of the population [11].

In this paper, we have designed and analyzed a method for predicting the remaining lifespan curve from the information of previous days, based on artificial neural networks. Specifically, the curve is predicted using data on movement between days and live *C. elegans* count. The movement information is introduced into the model using one image per day showing *C. elegans* location and the count is introduced as a vector. The method analyzed from which days the remaining survival curve can be predicted with sufficient reliability. One of the drawbacks of using artificial neural networks to predict the future lifespan curve is the difficulty of estimating prediction uncertainty. Therefore, this work also analyzes whether the method can measure the confidence in the predictions of the model.

In summary, the contributions of this article are as follows:

• To propose a method for predicting lifespan curves in *C. elegans* assays using a bimodal neural network trained with synthetic data.
• Propose a method for estimating uncertainty in model predictions.
• Define and analyze a criterion based on a statistical test which can help to decide when the predictions are more reliable and can therefore be considered in order to halt the assay.

The remaining sections of this article are structured as follows: Section 2 shows the proposed lifespan prediction method. Section 3 presents the experiments and results. In Section 4 ablation studies are presented. Section 5 contains the discussion and conclusion.

## 2 Methodology

**Fig. 1:**
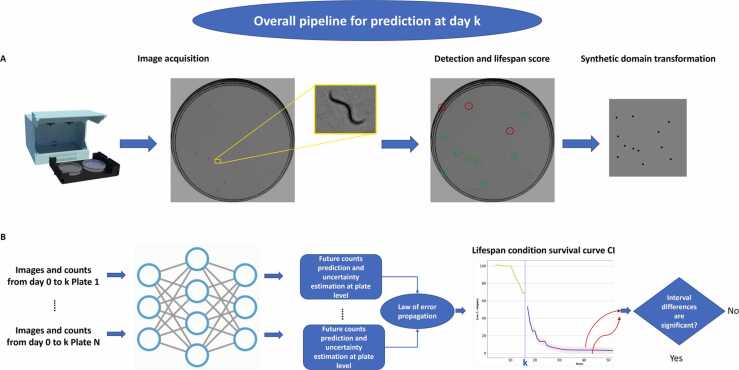
General outline of the proposed method.

In this paper a prediction method is proposed to complete lifespan curves using data taken from the beginning of the experiment (images and live counts) to the current day (day k). Fig. 1 shows a general outline of the method. The first step consists of capturing the images using a monitoring system, and detecting and counting live/dead nematodes on that day, either manually or using automatic methods. The captured image is transformed to the synthetic domain on which the deep learning model has been trained. The use of this synthetic domain allows the model to be trained with a simple simulator and avoids the labeling of large datasets. As the *C. elegans* population of the lifespan per condition experiment is divided into several test plates, the survival curves of each plate are obtained and then added up to obtain the count per condition. For each plate, the prediction is made using the deep learning model and the uncertainty is estimated. Subsequently, the law of error propagation is used to calculate the uncertainty in the prediction of the count per condition. Using this uncertainty, a confidence interval is calculated. The last step of the method consists of performing a statistical analysis (log-rank test) to determine whether the differences between the confidence interval limits are significant. If the differences are not statistically significant, the predictions of the method can be considered more reliable and can, therefore, be considered in order to halt the experiment.

### 2.1 Image capture method and input generation

Different capture systems are available to capture images of the whole Petri dish [12], [13], [14], [15], [16]. In our case, we use the devices developed in our laboratory [17], [18]. Images were captured using the SiViS [17] monitoring system, which uses intelligent lighting control to obtain images of the whole Petri dish. The captured images are used to detect and count live and dead *C. elegans* by manually analyzing the images or using automatic techniques [19], [20], [21]. The real image is transformed to a synthetic domain image, on which the neural network is trained.This synthetic domain image consists of drawing circles on a gray background at the nematode locations. In our case, we use automatic methods developed in previous work. Specifically, these are detection networks [21] and a live/dead classifier [20]. With these algorithms, the centroid coordinates of all *C. elegans* on the plate and their classification as live or dead are obtained. The results provided by the automatic methods were manually checked to avoid errors. Using this synthetic domain allows the network to be trained with a simple simulator without the need for labeling, and also enables the use of images captured with different acquisition systems.

### 2.2 Bimodal neural network architecture

**Fig. 2:**
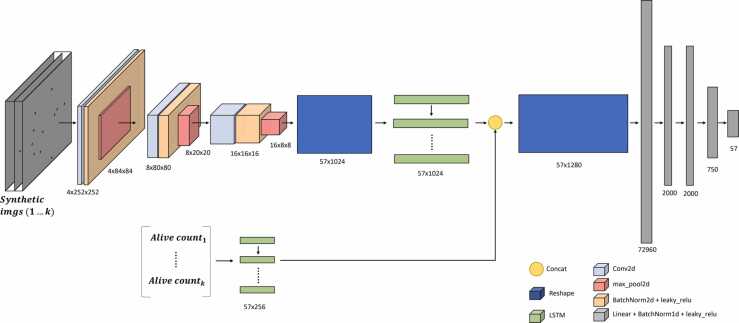
Diagram showing the deep learning model used.

The proposed method of predicting lifespan curves is a time series prediction. Future values are predicted using the information captured up to the current day. The information available up to the current day are the live *C. elegans* count values and the images, which provide information about nematode activity (displacement between days). These images are a simplification obtained from the real ones. On a constant background, circular blobs are drawn at the positions where the *C. elegans* are located. The proposed model is therefore a bimodal input network (numerical data and images). In the proposed model, on the one hand, a CNN-LSTM network extracts the spatiotemporal features and, on the other hand, a LSTM encodes the time series of the count. These features are concatenated and finally a fully connected network performs the regression to obtain the future counts. A diagram showing the prediction method can be seen in Fig. 2. The details of the architecture used are shown in Table A.1.

### 2.3 Uncertainty estimation

Several methods are available for estimating uncertainty for predictions with artificial neural networks, such as Bayesian neural networks [22], [23]. These methods have the disadvantage of requiring modifications in the model and in the learning process, in addition to increasing the computational cost. In this work we have chosen to approximate the uncertainty by estimating the variance of the output by introducing Gaussian noise in the input. The variable for which uncertainty is desired is *C*, the count of the number of live *C. elegans* on each day of the lifespan test. Therefore, *C* (Eq. (1)) is a function of *P*_1_, *P*_2_, …, *P*_*k*_

(1) $$C=f\left(P_{1},P_{2},\ldots ,P_{k}\right)=P_{1}+P_{2}+\cdots +P_{k}$$

where *P*_1_, *P*_2_, …, *P*_*k*_ are the counts of each plate that make up the condition. The uncertainty (variance) in *C* can be approximated using the law of error propagation (Eq. (2)):

(2) $$S_{C}^{2}={\left(\frac{\delta C}{\delta P_{1}}\right)}^{2}\cdot S_{P_{1}}^{2}+{\left(\frac{\delta C}{\delta P_{2}}\right)}^{2}\cdot S_{P_{2}}^{2}+\cdots +{\left(\frac{\delta C}{\delta P_{k}}\right)}^{2}\cdot S_{P_{k}}^{2}$$

Hence, the approximation of the standard deviation is (Eq. (3)):

(3) $$S_{C}=\sqrt{{\left(\frac{\delta C}{\delta P_{1}}\right)}^{2}\cdot S_{P_{1}}^{2}+{\left(\frac{\delta C}{\delta P_{2}}\right)}^{2}\cdot S_{P_{2}}^{2}+\cdots +{\left(\frac{\delta C}{\delta P_{k}}\right)}^{2}\cdot S_{P_{k}}^{2}}$$

where $S_{C},S_{P_{1}},S_{P_{2}},S_{P_{k}}$ are the standard deviations of the condition and plate counts respectively. And $\frac{\delta C}{\delta P_{1}},\frac{\delta C}{\delta P_{2}},\ldots ,\frac{\delta C}{\delta P_{k}}$ are the partial derivatives of the function with respect to each of the variables.

Since *C* linearly depends on each of *P*_1_, *P*_2_, …, *P*_*k*_, the partial derivatives are equal to 1, leaving the expression reduced (Eq. (4)):

(4) $$S_{C}=\sqrt{S_{P_{1}}^{2}+S_{P_{2}}^{2}+\cdots +S_{P_{k}}^{2}}$$

Consequently, to obtain the uncertainty in the analysis for each condition, it is necessary to know the standard deviation in the model predictions (per plate). Given an input, the model returns a single output, so uncertainty cannot be obtained directly. One way to estimate uncertainty in the model output is to generate new inputs from the original by adding noise. If the input is a vector *X* (Eq. (5)):

(5) $$X=\left\{x_{1},x_{2},\ldots ,x_{d}\right\}$$

A noise *R* (Eq. (6)) is added:

(6) $$R=\left\{r_{1},r_{2},\ldots ,r_{d}\right\}$$

where *r*_*i*_ ∼ *N*(0, 1) is a random value obtained from a normal distribution of mean 0 and variance 1. Using this method (Algorithm 1), *N* new inputs are generated for each input. With these simulated inputs, *N* predictions are generated, which allow the uncertainty to be calculated as the mean (Eq. (7)) and standard deviation of these predictions (Eq. (8)).

(7) $$\overset{¯}{P_{k}}=\frac{\sum_{i}^{N}P_{i}}{N}$$

(8) $$S_{k}=\sqrt{\frac{\sum_{i}^{N}{\left(P_{ki}-\overset{¯}{P_{k}}\right)}^{2}}{N-1}}$$

Knowing the mean and standard deviation of the prediction of each plate we can find out the standard deviation of the prediction per condition *S*_*C*_ using the error propagation method (Eq. (4)). Once this is obtained, a confidence interval can be calculated (Eq. (9)):

(9) $$CI=\left[\overset{¯}{C}-Z_{\frac{\alpha }{2}}\cdot \frac{S_{C}}{\sqrt{n}},\overset{¯}{C}+Z_{\frac{\alpha }{2}}\cdot \frac{S_{C}}{\sqrt{n}}\right]$$

where $Z_{\frac{\alpha }{2}}$ is the critical value such that $P\left(x\leq Z_{\frac{\alpha }{2}}\right)=1-\frac{\alpha }{2}$ and *n* is the number of samples (plates).

Algorithm 1 Uncertainty estimation for Model prediction.

**Table:**

### 2.4 Halting criteria

Applying Eq. (9), a confidence interval is calculated for the count of each day of the experiment. With the extremes of the confidence intervals for all days, two survival curves (upper and lower limit) are generated. A log-rank statistical test is applied to these generated lifespan curves to determine whether the differences between them are statistically significant using the open-source software OASIS [24]. Taking a significance level (alpha) of 5 %, if the calculated *p-value* is greater than alpha, the null hypothesis is accepted, and it is concluded that there are no significant differences between the upper and lower limit curves, and therefore it could be considered a good time to end the trial.

### 2.5 Neural network training method

#### 2.5.1 Simulator

The training of the neural network was performed using a simulator, which generates synthetic type images, as shown in Fig. 2. This simulator generates lifespan curves using the 2-parameter Weibull model [25], in which the one-day survival function is calculated using (Eq. (10)).

(10) $$survival\left(t\right)=e^{\frac{-t}{b}a}$$

Where the parameters (*a* and *b*) are related to the slope of the mortality curve and the mean lifespan, respectively.

**Fig. 3:**
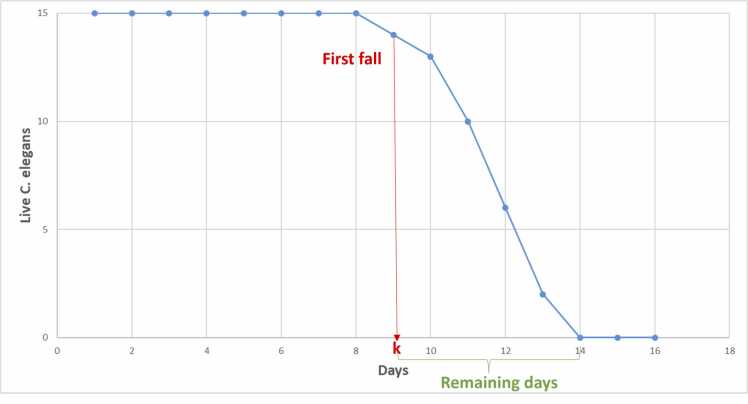
Training data generation scheme. First, a lifespan curve (red curve) is generated. For each curve, as many data are obtained as days remaining from the first drop to the end.

**Fig. 4:**
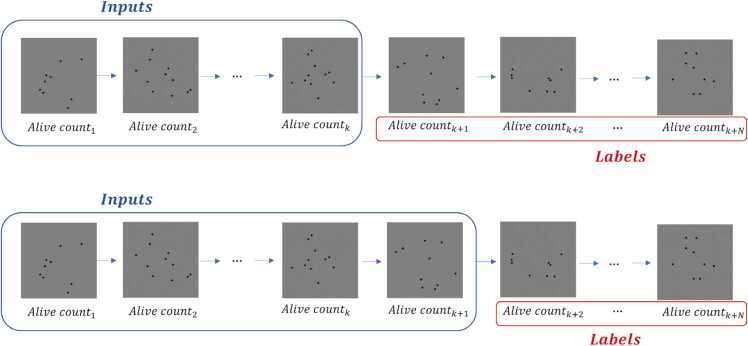
Generating inputs and labels. Starting from the day of the first fall of the curve (day k) the number of inputs is increased, thus generating one input for each remaining day.

The simulator parameters are the number of nematodes to be simulated and the values of the parameters *a* and *b*. Using these values, it is iterated from day to day by calculating the % survival until it reaches zero. The generated Weibull curves are modified with transformations that randomly add certain distortions present in the real data (no capture days). Adding the randomness present in the real data to the synthetic data facilitates generalization, preventing overfitting. Based on the curve obtained, the images are generated by randomly positioning the circles, taking into account that when a *C. elegans* dies, its position no longer varies. Each training data is a combination of the simulator parameters (number of *C. elegans*, mean lifespan, slope) and the input sequence length. The minimum sequence length is from the first day of capture to the first day the curve falls, i.e., the first day a *C. elegans* dies. For each curve, as many data are obtained as there are remaining days from the first drop to the end, as seen in Fig. 3. Fig. 4 shows this input and label generation procedure. The synthetic dataset has a total of 93024 data points, of which 80 % was used for training and 20 % for validation.

#### 2.5.2 Hyperparameters

The network was implemented and trained using the Pytorch deep learning framework [26] on a computer with an Intel® Core™ i7–7700 K processor and NVidia GeForce GTX 1070 Ti graphics card. The model was trained for 100 epochs with a learning rate of 0.001 and a batch size of 16 samples. The mean squared error (MSE) was used as the cost function (Eq. (11)) and the optimizer used was SGD.

(11) $$MSE=\frac{1}{N}\overset{N}{\underset{i=1}{\sum }}{\left(\overset{ˆ}{y_{i}}-y_{i}\right)}^{2}$$

Where *N* is the total number of frames in the batch, $\overset{ˆ}{y_{i}}$ and *y*_*i*_ are the model prediction for the number of live *C. elegans* in frame *i* and label *i* respectively.

Training and validation curves (Fig. A.1) are included in Appendix A.

## 3 Experiments and results

Manually labeled data from real experiments captured with the SiViS system were used to analyze and validate the proposed prediction method. This section explains the conditions under which the experiments were performed, the validation methods as well as the results obtained.

### 3.1 *Caenorhabditis elegans* strains and culture conditions

Two different strains of *C. elegans*, N2, Bristol (wild-type), and CB1370, *daf-2* (*e1370*) obtained from the Caenorhabditis Genetics Centre at the University of Minnesota, were used. Nematodes were maintained at 20^∘^C in 55 mm Petri dishes under the conditions described in [27].

Age-synchronized adult worms were transferred to Petri dishes containing Nematode Growth Medium (NGM). *Escherichia coli* (OP50) was used as a food source and was placed in the center of the plate to avoid cases of escape. In cases where a nematode escapes or hides in the agar, it is considered dead if it does not reappear in the following days. FUdR (0.2 mM) was added to prevent reproduction and Fungizone to reduce contamination.

A laboratory technician was in charge of capturing images daily during the assay. To do this, the plates were removed from the incubator, placed in the capture system for a sequence of 30 images to be taken at 1 fps and then returned to the incubator. Our acquisition systems have a plate fixation system that allows us to prevent shifting and place the plates in the same position every day [17].

Once the images were captured, they were analyzed and the count of live and dead *C. elegans* was obtained on each day of the assay. The criteria followed for counting was based on movement analysis of the *C. elegans* during the 30-image sequence. Nematodes that moved during the sequence were counted as alive. If any nematode remained motionless, it was searched for in the images of the previous and subsequent day. If it varied its position and posture, it was considered alive or, otherwise, it was considered dead.

### 3.2 Validation dataset

Two assays were performed with *C. elegans* N2 to which life-extending substances (metformin was used at 50 mM) were added under two different conditions, each consisting of 10 plates containing 10–15 worms each. An assay was also performed with *C. elegans* N2 with the normal mean life (approximately 14 days) composed of four conditions each with four plates containing between 10 and 15 nematodes. On the other hand, three experiments were performed with *C. elegans* strain *daf-2*, each with four plates containing between 10 and 15 nematodes. In this way we could validate the method with strains of short, medium and long lifespan.

### 3.3 Analysis of the day on which the experiment is halted

In this validation experiment, the proposed method was applied to real manually labeled assays. Starting on the first day of curve fall, the number of inputs was increased (as shown in Fig. 4) until the halting criterion determined that the differences between the extremes of the confidence interval were not statistically significant.

Different indicators were used to evaluate the method: (1) the day on which the method proposes halting the trial was compared with the mean lifespan; (2) the MAE (%) between predicted and manually labeled series was calculated. For this purpose, the survival percentage was calculated for each predicted day using Eq. (12) and then the average of the difference in absolute value between the predicted value and the labeled one was obtained (Eq. (13)); and (3) finally, the method analyzed whether the differences between the two were statistically significant using the log-rank test with the OASIS software.

(12) $$\%\mathrm{live}C.elegans=\frac{\mathrm{live}\;C.elegans\;\text{current day}\;\cdot 100}{\mathrm{initial}\;\mathrm{live}\;C.elegans}$$

(13) $$\mathrm{MAE}=\frac{\sum_{d=1}^{days}|\%livemanual\left(d\right)-\%liveautomatic\left(d\right)|}{days}$$

**Fig. 5:**
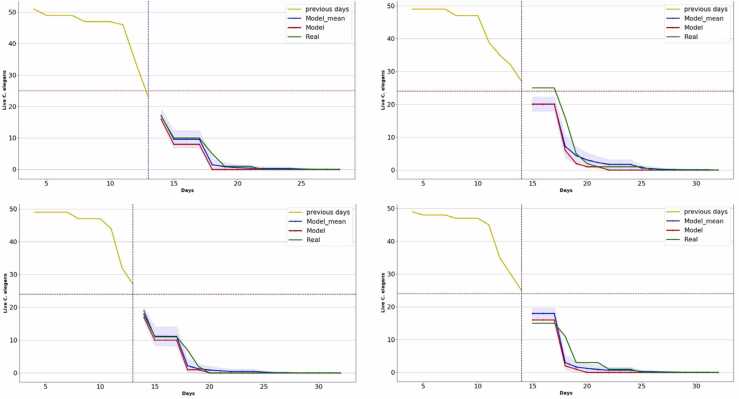
Lifespan curves obtained with the prediction method in the short mean lifespan assays. The horizontal axis shows the days of the experiment, and the vertical axis shows the number of live *C. elegans*. The yellow curve represents the input data to the model. The blue curve is the mean of the predictions together with its confidence interval. The red line is the neural network prediction and the green line is the manually labeled curve.

Results obtained with strain N2 with the normal mean lifespan assays are as follows (Table 1 and Fig. 5):

**Table 1:** Results of experiments performed with strain N2 with the normal mean lifespan.

Exp	Mean lifespan	Mean lifespanNN	Day halted	MAE (%)	P-value
ScA	13.75	13.45	13	3.27	0.39
ScB	15.69	15.02	14	5.61	0.12
ScC	14.22	13.98	13	3.5	0.43
ScD	14.96	14.61	14	4.27	0.25

The results in Table 1 show that in all assays the method proposes halting the experiment approximately on the same day that the mean lifespan is reached, and in all cases the statistical test determines there are no statistically significant differences (*p-value>0.05*) between the manually labeled curve and the prediction.

**Fig. 6:**
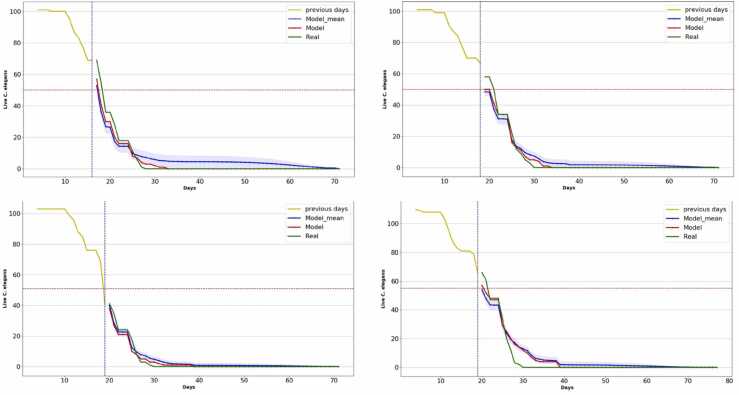
Lifespan curves obtained with the prediction method in the medium mean lifespan assays. The horizontal axis shows the days of the experiment, and the vertical axis shows the number of live *C. elegans*. The yellow curve represents the input data to the model. The blue curve is the mean of the predictions together with its confidence interval. The red line is the neural network prediction and the green line is the manually labeled curve.

The results obtained with the N2 strain assays in which life-extending substances are added are as follows (Table 2 and Fig. 6):

**Table 2:** Results of experiments performed with N2 strain assays in which life-extending substances are added.

Exp	Mean lifespan	Mean lifespanNN	Day halted	MAE (%)	P-value
M1cA	18.64	18.24	16	4.02	0.51
M1cB	20.2	20.07	18	3.03	0.88
M2cA	19.61	19.51	19	2.23	0.81
M2cB	20.85	21.51	19	5.36	0.15

The results in Table 2 show that, in all assays, the method proposes halting the experiment either before or on the same day that the mean lifespan is reached, and in all cases the statistical test determines there are no statistically significant differences (*p-value* *>* *0.05*) between the manually labeled curve and the prediction.

**Fig. 7:**
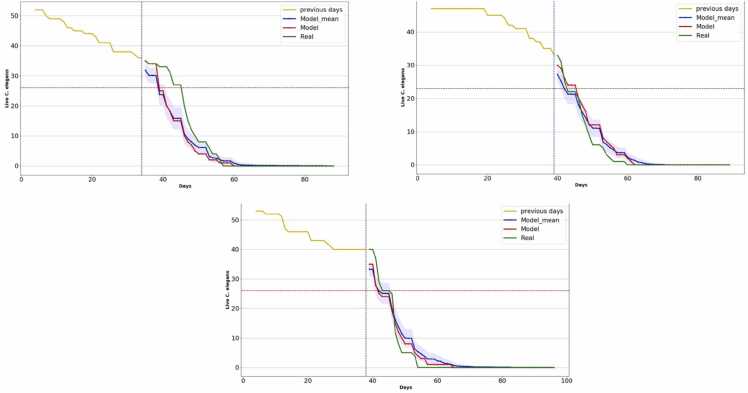
Lifespan curves obtained with the prediction method in the long mean lifespan assays. The horizontal axis shows the days of the experiment, and the vertical axis shows the number of live *C. elegans*. The yellow curve represents the input data to the model. The blue curve is the mean of the predictions together with its confidence interval. The red line is the neural network prediction and the green line is the manually labeled curve.

Results obtained with *C. elegans* strain *daf-2* assays are showed in Table 3 and Fig. 7:

**Table 3:** Results of experiments performed with *C. elegans* strain *daf-2*.

Exp	Mean lifespan	Mean lifespanNN	Day halted	MAE (%)	P-value
LcA	38.58	36.21	34	9.54	0.09
LcB	42.3	43.45	39	5.92	0.25
LcC	39.28	39.38	38	5.24	0.75

The results in the table show that in all trials the method proposes halting the experiment either before or on the same day that the mean lifespan is reached, and in all cases the statistical test determines there are no statistically significant differences between the manually labeled curve and the prediction.

### 3.4 Comparison with a curve-fitting method

In this experiment we compared the error between fitting the curve to a parametric model (Weibull) and the prediction made by the neural network model on the day when the method determined the prediction was reliable. To make this comparison, the inputs from the count (from the beginning to the current day) were used and the *curve_fit* function from the SciPy [28] library was used to fit the curve. The MAE (%) between the prediction and the manual labels is calculated from the day the experiment stops to the end, i.e., the future period. The previous period is known; therefore, it is not taken into account to calculate the error.

As can be seen in the Table 4, Table 5, Table 6 , the prediction made with the model obtained better results than the adjustment of the parameters of the Weibull model in all the experiments.

**Table 4:** Results of experiments performed with strain N2 with the normal mean lifespan.

Experiment	MAE (%)_NN	MAE (%)_Opt
ScA	3.27	5.23
ScB	5.61	10.03
ScC	3.5	3.79
ScD	4.27	7.42

**Table 5:** Results of experiments performed with N2 strain assays in which life-extending substances are added.

Experiment	MAE (%)_NN	MAE (%)_Opt
M1cA	4.02	8.27
M1cB	3.03	4.68
M2cA	2.23	3.47
M2cB	5.36	7.32

**Table 6:** Results of experiments performed with *C. elegans* strain *daf-2*.

Experiment	MAE (%)_NN	MAE (%)_Opt
LcA	9.54	20.44
LcB	5.92	10.74
LcC	5.24	36.1

## 4 Ablation studies

### 4.1 Modal contribution analysis

This experiment seeks to analyze the need to use a bimodal model. For this purpose, the results obtained with unimodal networks (only images and only counts) have been compared with the proposed bimodal network (images and counts). Figs. A.2–A.4 show the diagrams of the models used. The models were trained using the hyperparameters mentioned in Section 2.5. The comparison has been performed by calculating the MAE in the same way as in Section 3.3, i.e., on the day when the stopping criterion determines that the assay can be terminated. As the initialization of the weights is randomized in each training, the trainings have been repeated three times each. Subsequently they have been validated and mean and standard deviation have been obtained. The results obtained are shown in the following Table 7:

**Table 7:** Ablation study on modal contribution. MAE (%) of the 3 replicates of the experiment, as well as the mean and standard deviation are presented.

Method	Exp 1	Exp 2	Exp 3	Mean	Std
Only images	9.62	9.02	8.58	9.07	0.52
Only counts	8.05	7.73	7.30	7.69	0.38
Counts + Images	4.73	6.61	6.19	5.84	0.99

The results show that the neural network with only images performs worse predictions than the network with only counts. Merging the two modes with the bimodal method gives the best results.

### 4.2 Analysis of the introduction of movement information

In this experiment we have compared two alternative ways of entering the movement information of the worms between days. The first option (Fig. A.5) is to enter each day’s information as a matrix of size 16 × 2 in which the coordinates (X, Y) of the worms are stored. These coordinates are always ordered in the same way (from lowest to highest using X as the primary key and Y as the secondary key). The matrix has 16 rows because this is the maximum number of *C. elegans* considered. If there are less than 16 worms, empty positions in the matrix are filled with zeros. The second alternative proposed is to use an image with a constant background and draw circles at the position of the worms.

The models were trained using the hyperparameters mentioned in Section 2.5. The comparison has been performed by calculating the MAE in the same way as in Section 3.3, i.e., on the day when the stopping criterion determines that the assay can be terminated. The trainings have been repeated three times each and mean and standard deviation have been obtained Table 8:

**Table 8:** Ablation study on introduction of movement information. MAE (%) of the 3 replicates of the experiment, as well as the mean and standard deviation are presented.

Method	Exp 1	Exp 2	Exp 3	Mean	Std
Counts + Coordinates	7.60	7.56	7.38	7.51	0.12
Counts + Images	4.73	6.61	6.19	5.84	0.99

The results obtained with the bimodal method with images are better than those obtained with the method with coordinates. Theoretically the methods are equivalent, the difference may be in the adjustment of hyperparameters, such as the batch size.

## 5 Discussion and conclusions

A method has been proposed to predict the evolution of the *C. elegans* lifespan curve from the data gathered on the first days of the assay. The method employs a bimodal artificial neural network that receives two sequences as inputs: (1) an image for each day from the beginning of the assay until the current day with the positions of the nematodes; (2) the count of the number of live *C. elegans* until the current day.

There is no fixed length at which prediction can be made that guarantees reliable results. For this reason, it was necessary to provide a measure of prediction reliability to assist the researcher in decision making. The deep learning model does not directly provide a measure of prediction confidence, so a method was assessed for estimating uncertainty based on the variance of the output when noise is introduced into the input. Using this uncertainty estimate, a criterion for deciding whether the prediction is reliable has been analyzed.

The model was trained with simulated data, generating the lifespan curves from the parametric Weibull model. This has avoided the temporal and economic cost of acquiring and labeling a dataset large enough to train the model. The approximate cost of labeling is approximately 13 working days per condition for a 30-day assay. To this cost must be added the preparation time of the assay plates and the capture days which vary between 30 and 60 days depending on the strain (N2 and *daf-2*). In our case, we performed eight lifespan curves with the N2 strain and three with the *daf-2* strain. The total cost of creating the dataset represented approximately 1 year and a half.

Moreover, since the images generated by the simulator are of simple synthetic domain, the method can be used with images captured using different monitoring systems. If we had used real images captured with our system, the method could not be used with capture systems differing from those of our model.

The results obtained in Section 3.3 show that the method makes predictions it considers reliable either a couple of days before reaching the mean lifespan or on the same day as the mean lifespan. These results were to be expected since uncertainty is very high during the first days and therefore the predictions are unreliable until the middle of the assay is reached. It has also been shown in Section 3.4 that the prediction obtained has less error than trying to fit the curve to the parametric Weibull model using parameter optimization.

Generally speaking, the results are good; however, the method has some limitations. The prediction has to be started from the first day the curve falls in all the plates comprising the condition from which the total count is to be obtained. If this is not taken into account, it can lead to errors in the uncertainty estimation and give a high confidence interval in a prediction where it is unfounded.

Another limitation of our method is the capture system used. As it requires an operator for the daily placement of the plates, thus no data are recorded on holidays, generating steps in the lifespan curve. If we were to employ another automatic capture device such as Lifespan Machine [13] the results of the proposed method could be improved by having more points available to predict future lifespan. Regarding the applicability of the method, it should be noted that our method is designed to perform the lifespan test without transference between plates to replace agar. This is because we use the information of variation in the position of the worms from day to day to determine whether they are alive or dead. The model was trained with plates on which there were between 10 and 15 worms with a mean lifespan between 10 and 57 days. In order to apply the method to other capture systems and other strains the model would have to be retrained with the new ranges.

In the manually obtained curves we can observe that the data do not fit perfectly to a Weibull distribution. This causes some values of the real curves to fall outside the confidence interval provided by the method. This discrepancy between the parametric curves and those of the real assay may be due to the following reasons: (1) as discussed in [29], the parametric models assume that in one condition the aging of nematodes is homogeneous. However, in most assays it is observed that it is heterogeneous, i.e., there are subpopulations that age differently; (2) possible errors in the input values entered into the model, when there are detection errors (e.g., occlusions at the edges of the Petri dish). If working with few *C. elegans* per plate, as in our case, small variations can lead to a less accurate prediction; and (3) the different stages observed in the curves because no images are captured on weekends and the count of the last available day is maintained.

This tool could be very useful in assays where numerous conditions have to be tested. It allows researchers to know the possible evolution of the lifespan tests before the end of the experiment. If the researcher decides to halt the experiment at approximately the mean lifespan, the test time would be reduced by half, and thus twice as many tests could be performed in the same time. This is therefore a decision-making support tool for researchers, which could be very interesting with future developments. Our method can be useful using an automated lifespan method (e.g., Lifespan Machine [13], Wormotel [15], The Tower [30]). It is an alternative to manual screening assays based on stress resistance, as the correlation between stress resistance and longevity is unclear. Several studies have shown that there are cases where stress resistance and longevity are correlated. However, cases have also been found where this is not so [31], [32].

In future work, we will seek to analyze the use of models that can reflect a heterogeneous lifespan, since parametric models assume homogeneous lifespan models and implement them in the simulator. Another prospect for improvement is to use more complex models with a larger number of inputs (measurements of other phenotypes). This would also involve re-evaluating the uncertainty estimation method.

## CRediT authorship contribution statement

**Antonio García-Garví:** Conceptualization, Methodology, Software, Data curation, Writing – original draft. **Pablo E. Layana-Castro**: Software, Visualization. **Antonio-José Sánchez-Salmerón:** Conceptualization, Methodology, Writing – original draft, Investigation, Resources, Project administration, Funding acquisition.

## Declaration of Competing Interest

The authors declare that they have no known competing financial interests or personal relationships that could have appeared to influence the work reported in this paper.

## Data Availability

We created a repository on github with a demo of our method: https://github.com/AntonioGarciaGarvi/CelegansLifespanPrediction (accessed on 12 September 2022). The code, components and the guidelines to build the monitoring system and the assembly description can be found in the repository https://github.com/JCPuchalt/SiViS.
