# Supplementary material for: Analysis of a C. elegans lifespan prediction method based on a bimodal neural network and uncertainty estimation
Source: Comput Struct Biotechnol J. 2022 Dec 29;21:655–64. doi: 10.1016/j.csbj.2022.12.033 (PMC9826930; doi:10.1016/j.csbj.2022.12.033)
Supplement: Supplementary file 1 — Supplementary material [file mmc1.pdf]

Appendix A. Supplementary data

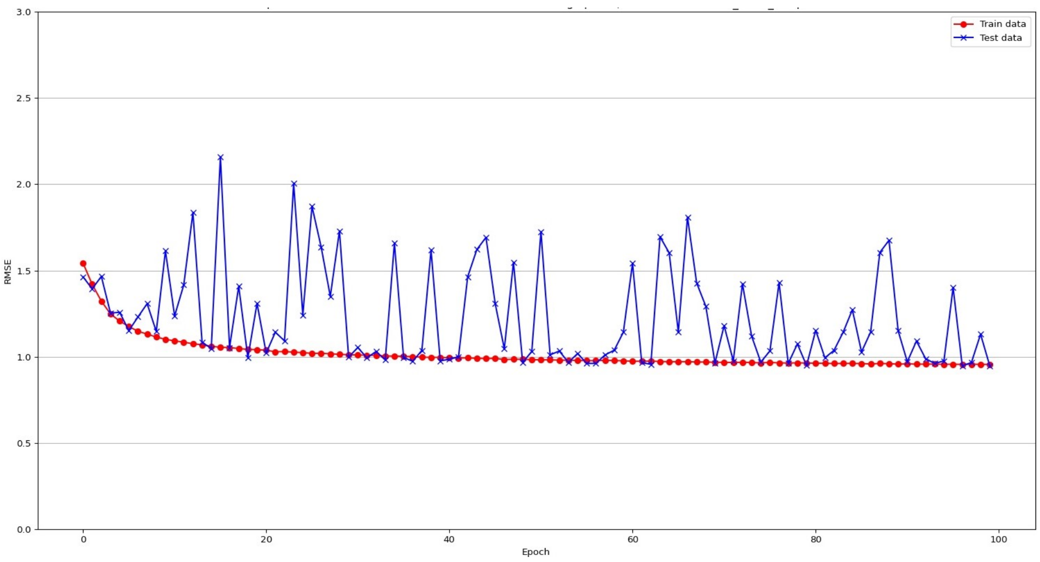

Figure A.1: Learning curves of the bimodal model. The X axis shows the number of epochs and the Y axis the RMSE loss value. The red curve corresponds to the training data and the blue curve to the test data.

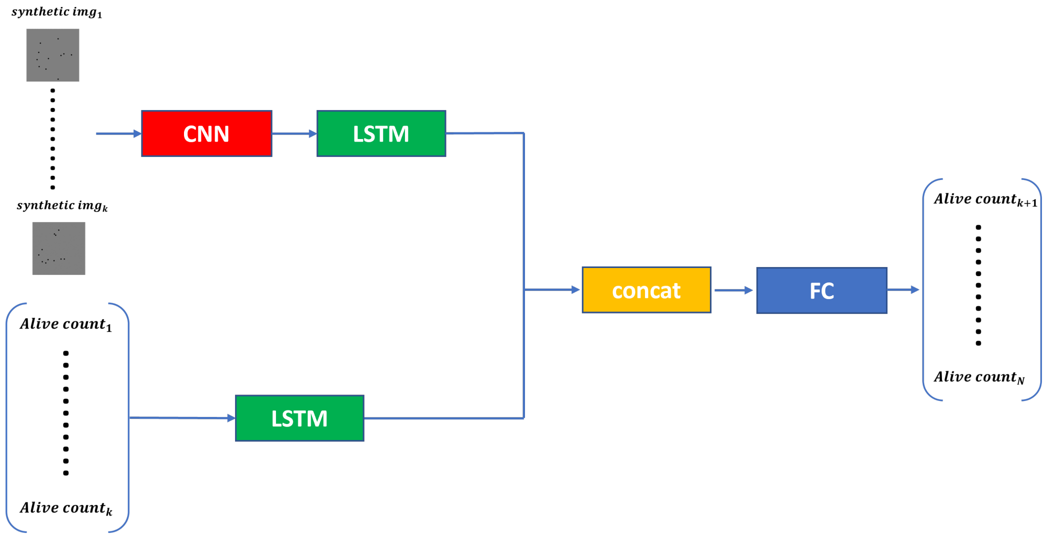

Figure A.2: Diagram showing the deep learning bimodal (images and counts) model used.

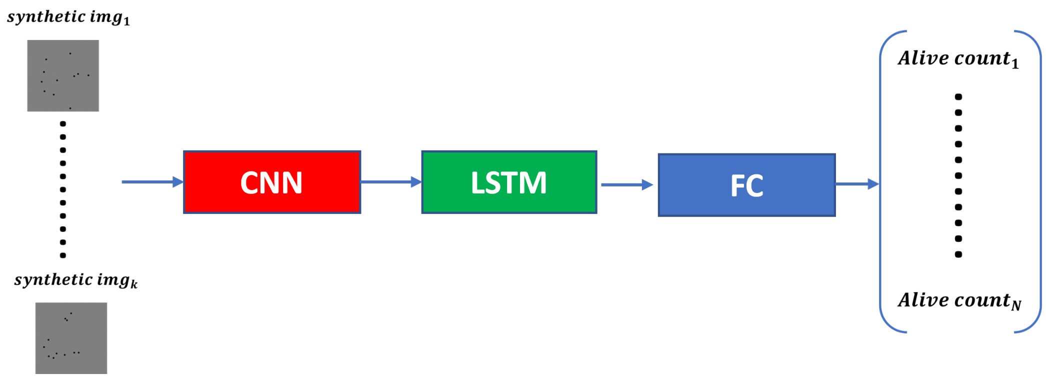

Figure A.3: Diagram showing the deep learning model using images as input.

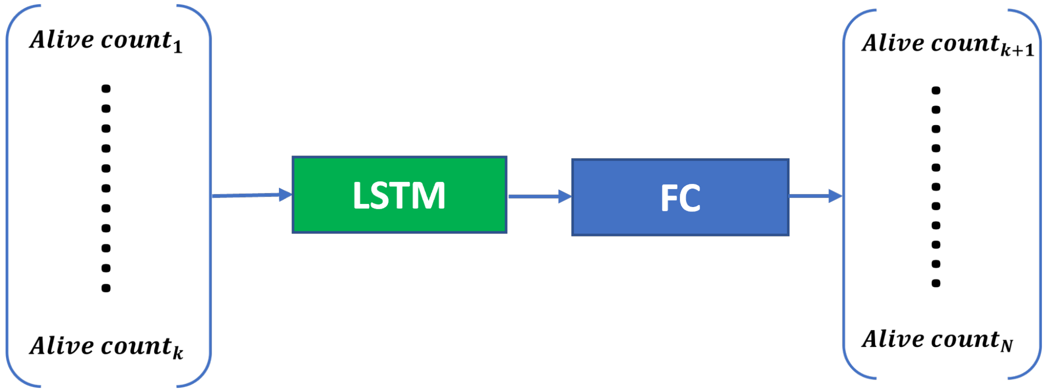

Figure A.4: Diagram showing the deep learning model using current counts as input.

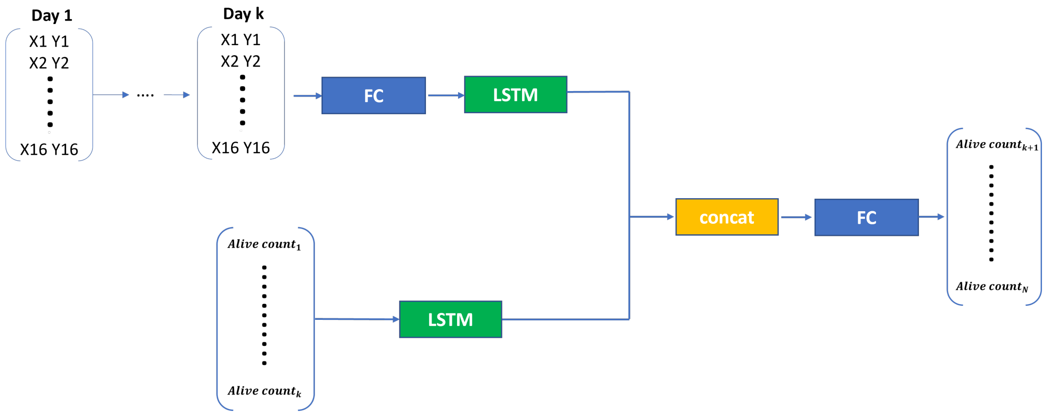

Figure A.5: Diagram showing the deep learning bimodal (coordinates and counts) model used.

Table A.1: Summary of the CNN–LSTM architecture used.The name of the layer, the output size of the layer and the details (number and size of cnn filters, hidden size of the lstm and size of the inputs and outputs of the linear layers) are shown.

Layer name	Output size	Layer details
conv1	[Batch size x seq length, 4, 252x252]	kernel size = 5x5, filters = 4, stride 1
cnn bn1	[Batch size x seq length, 4, 252x252]	$eps = 1e - 05$, momentum=0.1
leaky relu1	[Batch size x seq length, 4, 252x252]	-
max pooling1	[Batch size x seq length, 4, 84x84]	kernel size = 3x3, stride 3
conv2	[Batch size x seq length, 8, 80x80]	kernel size = 5x5, filters = 8, stride 1
cnn bn2	[Batch size x seq length, 8, 80x80]	$eps = 1e - 05$, momentum=0.1
leaky relu2	[Batch size x seq length, 8, 80x80]	-
max pooling2	[Batch size x seq length, 8, 20x20]	kernel size = 4x4, stride 4
conv3	[Batch size x seq length, 16,16x16]	kernel size = 5x5, filters = 16, stride 1
cnn bn3	[Batch size x seq length, 16, 16x16]	$eps = 1e - 05$, momentum=0.1
leaky relu3	[Batch size x seq length, 16, 16x16]	-
max pooling3	[Batch size x seq length, 16, 8x8]	kernel size = 2x2, stride 2
lstm	[Batch size, seq length, 1024]	In features = 1024<br>Hidden size = 1024<br>Num layers = 1
lstm2	[Batch size, seq length, 256]	In features = 1<br>Hidden size = 256<br>Num layers = 1
	Concatenation lstm - lstm2	
linear1	[Batch size, 2000]	In features = seq length x 1280<br>Out features = 2000
bn fc 1	[Batch size, 2000]	$eps = 1e - 05$, momentum=0.1
leaky relu fc1	[Batch size, 2000]	
linear2	[Batch size, 2000]	In features = 2000<br>Out features = 2000
bn fc2	[Batch size, 2000]	$eps = 1e - 05$, momentum=0.1
leaky relu fc2	[Batch size, 2000]	
linear3	[Batch size, 750]	In features = 2000<br>Out features = 750
bn fc 3	[Batch size, 750]	$eps = 1e - 05$, momentum=0.1
leaky relu fc3	[Batch size, 750]	
linear4	[Batch size, seq length]	In features = 750<br>Out features = seq length
